# Understanding metal-enhanced fluorescence and structural properties in Au@Ag core–shell nanocubes†

**DOI:** 10.1039/c9ra05103a

**Published:** 2019-09-17

**Authors:** Dae-Woong Jung, Jun Min Kim, Hyung Joong Yun, Gi-Ra Yi, Jung Young Cho, Haeun Jung, Gaehang Lee, Weon-Sik Chae, Ki Min Nam

**Affiliations:** Korea Basic Science Institute Daejeon 34133 Republic of Korea ghlee@kbsi.re.kr; Department of Chemical Engineering, Sungkyunkwan University Suwon 16419 Republic of Korea; Korea Institute of Ceramic Engineering and Technology Jinju 52851 Republic of Korea; Department of Chemistry and Chemistry Institute for Functional Materials, Pusan National University Busan 46241 Korea namkimin.chem@gmail.com; Analysis Research Division, Daegu Center, Korea Basic Science Institute Daegu 41566 Republic of Korea wschae@kbsi.re.kr

## Abstract

Au@Ag core–shell structures have received particular interest due to their localized surface plasmon resonance properties and great potential as oxygen reduction reaction catalysts and building blocks for self-assembly. In this study, Au@Ag core–shell nanocubes (Au@AgNCs) were fabricated in a facile manner *via* stepwise Ag reduction on Au nanoparticles (AuNPs). The size of the Au@AgNCs and their optical properties can be simply modulated by changing the Ag shell thickness. Structural characterization has been carried out by TEM, SAED, and XRD. The metal-induced fluorescence properties of probe molecules near the Au@AgNCs were measured during sedimentation of the Au@AgNCs. The unique ring-like building block of Au@AgNCs has dual optical functions as a fluorescence quencher or fluorescence enhancement medium depending on the assembled regions.

## Abstract 2

The unique ring-like building block of Au@AgNCs has dual optical functions as a fluorescence quencher and fluorescence enhancement medium.

## Graphical abstract

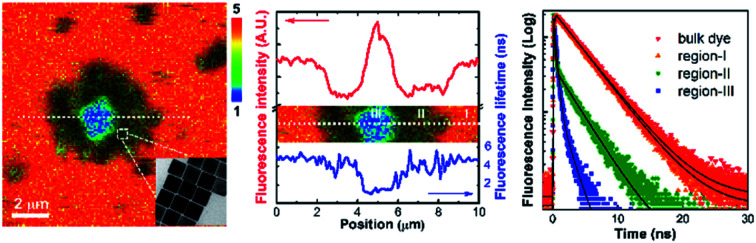

## Introduction

Combining Au and Ag into core–shell (Au@Ag) nanocrystals has attracted great interest due to the potential for exploiting these hybrids in catalysis,^1^ plasmonics,^2^ sensing,^3^ and imaging based on their optical properties. Numerous methods have been developed for synthesizing Au@Ag nanocrystals with different morphologies such as decahedrons, cubes, octahedrons, and rods.^4–7^ Specifically, Au@Ag nanocubes (Au@AgNCs) offer great potential as catalysts for oxygen reduction reaction^8^ and building blocks for self-assembly,^9^ and exhibit localized surface plasmon resonance properties.^10^ Several methods are now available for generating Au@AgNCs. However, simple synthesis of Au@AgNCs with controllable edge lengths over a wide range is still challenging.

Recently, it has been reported that plasmonic metal nanocrystals can modify the fluorescence characteristics of adjacent molecules.^11,12^ Understanding the spatial interactions between metals and nearby excited fluorophores is useful for the construction of models for fluorescence-based biological detectors and sensors. It is known that the excitation and emission processes of a fluorophore can be altered based on the proximity to metal nanocrystals. In one case, photoexcited electrons in fluorophores were rapidly trapped by a bound metal, resulting in fluorescence quenching.^13^ In another case, irradiation at a wavelength matching the specific surface plasmon resonance frequency of metal nanocrystals resulted in the accumulation of optical fields around the metal nanocrystals,^14^ hence reinforcing the absorption and enhancing the emission quantum yield of surrounding fluorophores.^15^ Metal enhanced fluorescence (MEF) has been widely studied as an efficient way to amplify fluorescence signals. Although the distance-dependence of the fluorescence enhancement and the quenching behavior have been widely studied, the factors that enhance MEF and the mechanism are still unclear.^11,12,16^

In this study, we describe a simple and efficient methodology for the aqueous synthesis of uniform and size-tunable Au@AgNCs. The synthesis route involves two sequential reactions, namely preparation of the Au NPs and stepwise Ag reduction on the Au NPs to afford Au@AgNCs. The metal-induced fluorescence properties of probe molecules near the Au@AgNCs are measured during sedimentation of the Au@AgNCs. The unique ring-like building block of Au@AgNCs has dual optical functions as a fluorescence quencher and fluorescence enhancement medium depending on assembled regions.

## Experimental

### Materials

The chemicals used in the synthesis of the spherical AuNPs include ethylene glycol (EG) (anhydrous, 99.8%), Au(iii) chloride trihydrate (49.0% metal basis), poly(diallyldimethylammonium chloride) (polyDADMAC) (*M*_w_ = 400 k–500 k, 20 wt% in H_2_O), and phosphoric acid (H_3_PO_4_) (85 wt% H_2_O). For synthesis of the Au@AgNCs, silver nitrate (AgNO_3_, 99.9999%), l-ascorbic acid (l-AA, 99%), and cetylpyridinium chloride (CPC, 99.9%) were needed. All chemicals were purchased from Aldrich and used without further purification. Ultrapure Millipore water with a resistivity greater than 18.2 MΩ cm was used in the preparation of the aqueous solutions.

### Synthesis of spherical AuNPs

For synthesis of the octahedral AuNPs, 0.4 mL of polyDADMAC and 0.8 mL of 1 M H_3_PO_4_ aqueous solution were added to 20 mL of EG solvent in a 50 mL glass vial. The mixture was stirred for 10 min at room temperature. Thereafter, 20 μL of 0.5 M Au(iii) chloride trihydrate aqueous solution was introduced into the mixture solution. The prepared gold precursor solution was loaded into an oil-bath preheated to 200 °C. In this process, the color of the solution changed from yellow to dark brown. The polyol reaction was allowed to proceed for 30 min, and the solution was then cooled to room temperature. Monodisperse spherical AuNPs seeds were prepared using octahedral AuNPs by an etching process. Au(iii) chloride trihydrate aqueous solution (5 μL; 0.5 M), as the etching reagent, was introduced into the solution of octahedral AuNPs, which was then allowed to stand for 24 h. Centrifugation of the solution at 22 000 rpm and redispersion of the precipitates in ethanol were repeated thrice to remove the excess reactants and byproducts. The purified monodisperse spherical AuNPs were dispersed in 1 mL of deionized (DI) water. From the UV/Vis spectrum of the gold solution and the Beer–Lambert equation, the concentration of the AuNPs was estimated to be 7.41 × 10^−10^ M.

### Synthesis of Au@AgNCs

For synthesis of the Au@AgNCs, 9.5 mL of DI water in a 50 mL glass vial was heated in an oil-bath, which was maintained at 60 °C for 30 min. Subsequently, 2 mL of 0.1 M CPC aqueous solution was added to the preheated DI water. After 10 min, 0.5 mL of the 7.41 × 10^−10^ M aqueous solution of spherical AuNPs was introduced into the mixture solution and allowed to react for 10 min. AgNO_3_ aqueous solution (50 μL of 0.05 M) and l-AA aqueous solution (25 μL of 0.5 M) were added to the mixture solution. After 6 h, the vial was cooled in cold water. Centrifugation at 15 000 rpm for 20 min and repeated washing with ethanol three times yielded the Au@AgNCs. The thickness of the Ag shell increased with increasing amounts of the AgNO_3_ and l-AA aqueous solutions.

### Characterization

TEM and EDX characterization of the morphology, crystal structure, and elemental composition of the samples were conducted with JEOL JEM-ARM200F and Bruker Quantax 400 instruments. X-ray diffraction data were collected with a Rigaku International Corporation MAX-2200 Ultima instrument for crystal structure analysis. SEM (S-4800, Hitachi) was employed to confirm the morphologies of the particles. A Shimadzu UV-2000 spectrophotometer was used for acquisition of the UV/Vis spectra for optical analysis. The silver content was analyzed by inductively coupled plasma spectroscopy (ICP, X-series, Thermo).

### Experimental details for fluorescence lifetime and imaging measurement

Fluorescence lifetime imaging was performed using an inverted-type scanning confocal microscope (MicroTime-200, Picoquant, Germany) with a 60 × water immersion objective. A single-mode pulsed diode laser (470 nm with ∼30 ps pulse width, 20 MHz repetition rate, and average power of ∼0.1 μW) was used as the excitation source. A dichroic mirror (490 DCXR, AHF), a long-pass filter (HQ500lp, AHF), a 50 μm pinhole, and a single-photon avalanche diode (PDM series, MPD) were used to collect emissions from the probe dye.

The standard probe, aqueous rhodamine 123 solution (1 × 10^−5^ M), was mixed with the Au@AgNC solution (∼4 × 10^−11^ M). The mixed solution, containing the dye and metal nanocrystals, was placed on a glass coverslip and put under the microscope. The Au@AgNCs sedimented to form aggregates. A time-resolved fluorescence confocal microscope was used to resolve the local fluorescence of the probe dye surrounding the metal nanocrystal aggregates. The time-correlated single-photon counting technique was used to count the fluorescence photons. FLIM images were recorded using the time-tagged time-resolved data acquisition technique. Exponential fitting of the fluorescence decay, extracted from the FLIM image, was performed using Symphotime software (version 5.3).

## Results and discussion

### Synthesis and characterization of Au@AgNCs

Synthesis of the Au@AgNCs involved two sequential reactions, namely preparation of the Au NPs and stepwise reduction of Ag on the Au NPs to afford Au@AgNCs. The super-spherical and monocrystalline gold nanoparticles (AuNPs) were generated by selective etching of the facets or edges of the octahedral AuNPs by exploiting their lower reduction potential.

The highly spherical AuNPs could provide a simple platform for precise research on optical engineering topics such as Fano-like resonance,^17^ extinction coefficient,^18^ and scattering.^19^ Therefore, along the same lines of previous studies, herein, spherical AuNPs were used as seed particles for synthesizing the Au@AgNCs.

**Fig. 1:**
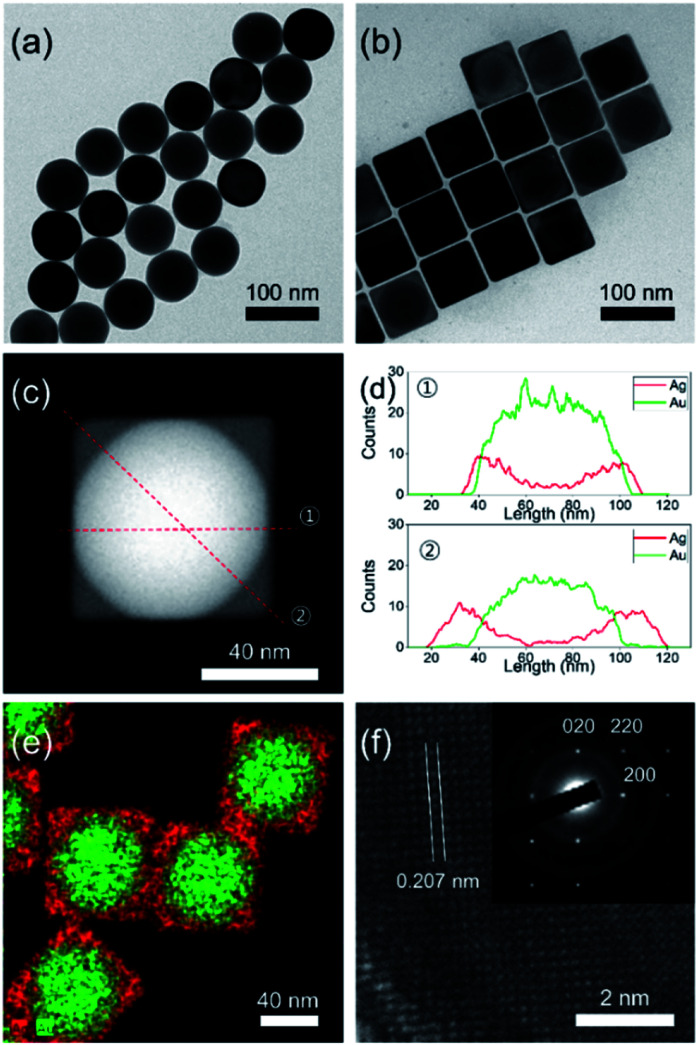
Bright-field TEM images of (a) spherical AuNPs and (b) Au@AgNCs. (c) Dark-field TEM image of a single Au@AgNC. (d) EDX line-profiles of a single Au@AgNC in directions ① and ② marked in (c). (e) EDX elemental mapping image of Au@AgNCs showing components of Au@AgNCs and structure. Au: green, Ag: red. (f) HRTEM image and SAED pattern (inset) of a single Au@AgNC.

Fig. 1a shows the transmission electron microscopy (TEM) image of the as-synthesized spherical AuNPs with an average diameter of 64.5 ± 2.9 nm, spherical shape, and high monodispersity. The seed-mediated route could allow the overgrowth of a cubic-type Ag shell on the spherical AuNPs. The experimental procedure from a prior study was modified for preparation of the Au@AgNCs.^1^ The addition of a silver precursor and l-ascorbic acid (l-AA) aqueous solution to the preheated solution of spherical AuNPs induced a gradual changed of the solution color from red to orange. TEM analysis of the resultant particles to elucidate their size, morphology, and crystal phase (Fig. 1b) showed that the nanoparticles had a cubic shape with an average edge length of 70.5 ± 3.2 nm, with high monodispersity. The relatively dark and bright areas in a single particle indicate the core@shell morphology of the nanoparticles. In order to confirm the morphology, careful TEM analysis was carried out in scanning TEM mode. The dark-field image in Fig. 1c clearly shows core@shell particles based on the sharp black-and-white contrast and the original shape of the spherical AuNPs as seed particles. Fig. 1d presents the energy dispersive X-ray (EDX) line profiles across a single Au@AgNC in two directions. The black-line of Ag increased in intensity from the outer wall toward the center, while the red-line for Au followed the opposite trend. The thickness of the Ag shell, which was determined from the gaps between the start position of Ag and Au, was 3 nm at line ① and 17 nm at line ②. The core@shell structure could be further verified by elemental mapping analysis, where gold is indicated by the green color and silver by the red color (Fig. 1e). The high-resolution TEM (HRTEM) image in Fig. 1f indicates a lattice spacing of 0.207 nm, corresponding to the (200) plane of the face-centered cubic (fcc) silver structure. The selected area electron diffraction (SAED) pattern in the inset of Fig. 1f shows several intense spots with square symmetry, which may correspond to a single crystal Au@AgNC bounded by the {100} facets of Ag.^20^

**Fig. 2:**
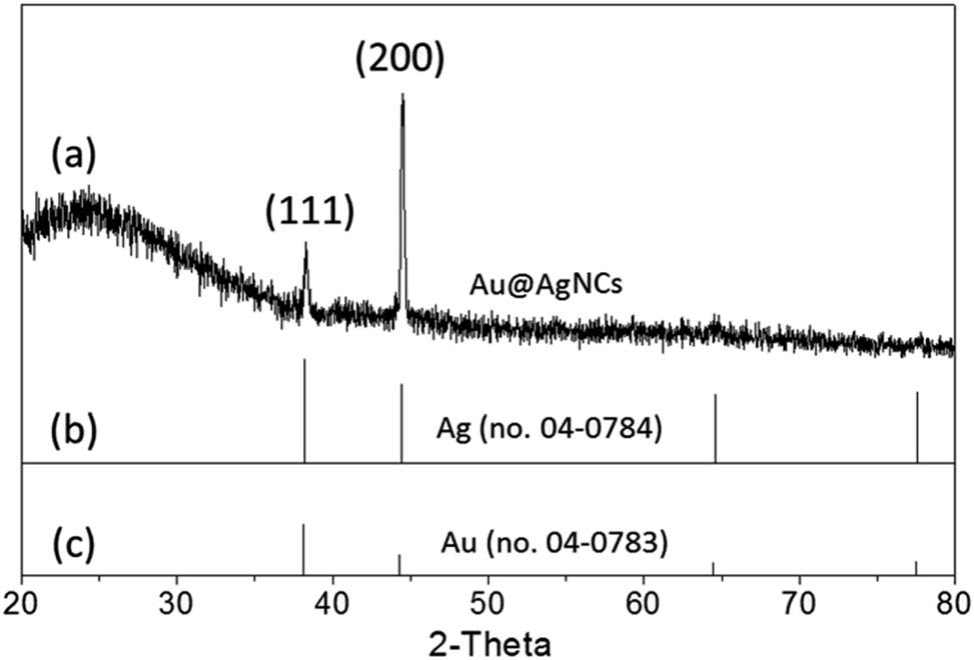
(a) XRD pattern of Au@AgNCs. The standard XRD patterns for (b) fcc Ag (JCPDS no. 04-0784) and (c) fcc Au (JCPDS no. 04-0783) are shown as bar diagrams at the bottom.

**Fig. 3:**
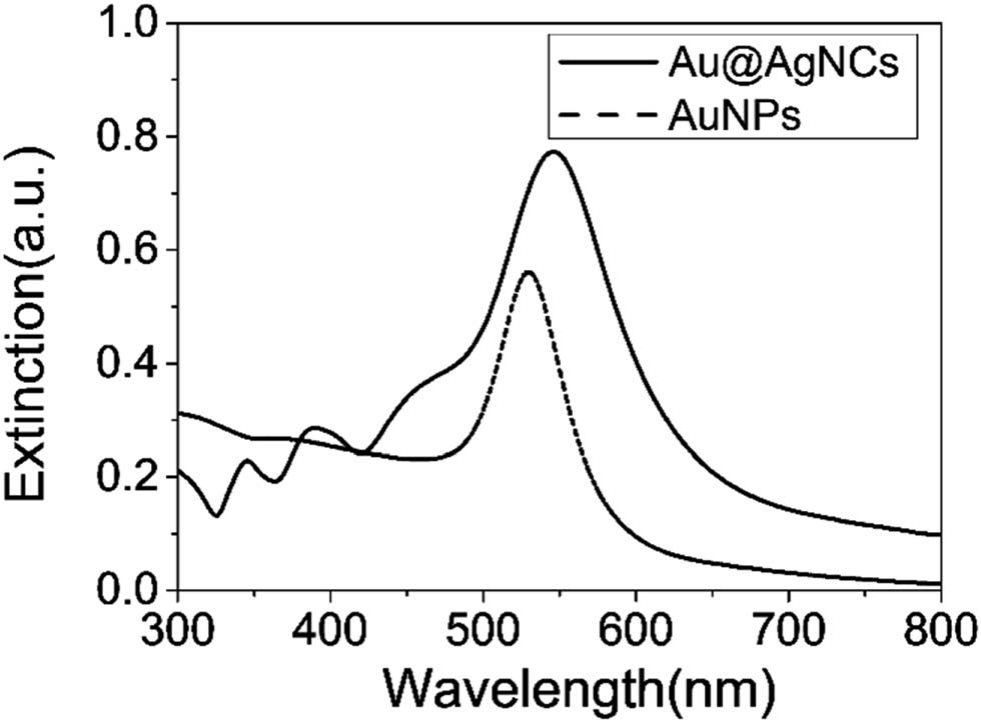
UV/Vis absorption spectra of spherical AuNPs (dotted line) and Au@AgNCs (solid line).

X-ray diffraction (XRD) patterns of the Au@AgNCs were acquired to evaluate the crystal phase (Fig. 2). The lattice constants of gold and silver in the face-center cubic structure (fcc) are very similar. The XRD signals of the Au@AgNCs were assigned to the (111) face of gold and (200) face of silver, consistent with the pattern of fcc Au (JCPDS no. 04-0783) and fcc Ag (JCPDS no. 04-0784), respectively. Frank–Van der Merwe mode shows a small lattice mismatch of 0.17% between fcc Ag and Au.^21^ Such mismatch can lead to epitaxial growth Ag on the surface of AuNP core in an experimental environment of available Ag reduction. Fig. 3 shows the UV/Vis absorption spectra of the spherical AuNPs and Au@AgNCs dispersed in 1 mL aqueous solution. The absorption spectrum of the Au@AgNCs reveals four characteristic peaks located at *ca.* 546, 345, 389, and 456 nm, representative of the Au core (546 nm) and the Ag shell (345, 389, and 456 nm), respectively.^22,23^

**Fig. 4:**
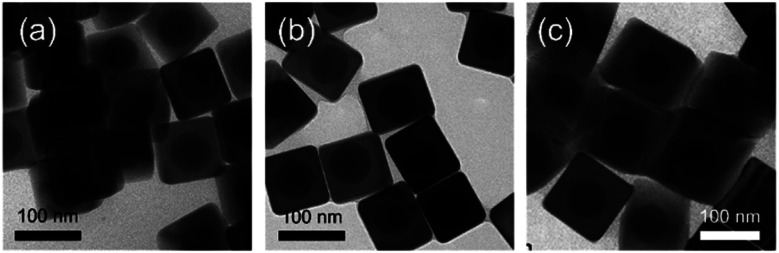
Bright-field TEM images of Au@AgNCs with average edge sizes of (a) 80.1 ± 3.5, (b) 93.3 ± 4.8, and (c) 122.4 ± 7.6 nm.

The size of the Au@AgNCs could be simply modulated by changing the Ag shell thickness. Au@AgNCs of various sizes with the same spherical AuNP cores were synthesized by addition of the Ag precursor. Fig. 4 shows the monodispersed Au@AgNCs with an average edge size of 80.1 ± 3.5, 93.3 ± 4.8, and 122.4 ± 7.6 nm, respectively. To observe Au@AgNC assembly characterization, 1 mL Au@AgNCs solution was put on top of the fluorine doped tin oxide (FTO) glass (Fig. S1†). After the solvent was completely evaporated, the Au@AgNC aggregates were examined using scanning electron microscopy (SEM). AuNP and Ag nanoparticles (AgNP) were also deposited using identical procedure to Au@AgNC assembly process, except for the use of Au and Ag samples. Fig. S1† shows the SEM images of the Au@AgNCs, AuNPs, and AgNPs.

Au@AgNCs and AgNPs showed many kinds of aggregates such as ring-like building block, and close-packed structure (Fig. S1a and c†). On the other hand, AuNPs are always well dispersed with close-packed structure on the substrate (Fig. S1b†).

### Fluorescence lifetime imaging analysis of Au@AgNCs

**Scheme 1:**
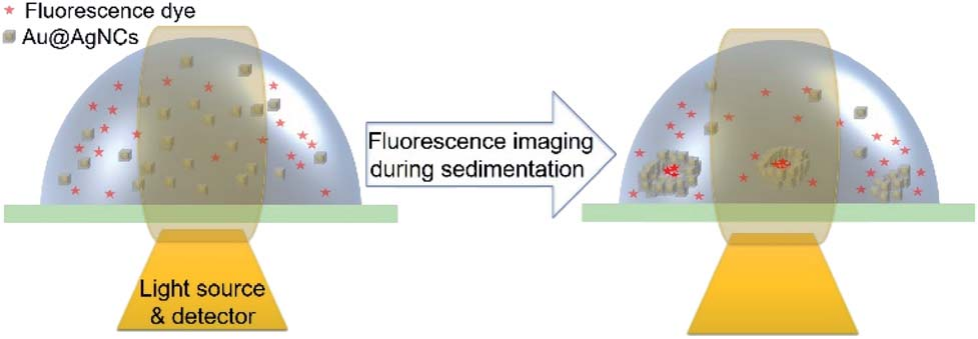
Schematic image of the metal-induced fluorescence of rhodamine 123 dyes during sedimentation of Au@AgNCs using 470 nm excited laser.

Fluorescence lifetime imaging microscopy (FLIM) was implemented using an inverted-type scanning confocal microscope. The standard probe, *i.e.*, aqueous rhodamine 123 solution (1 × 10^−5^ M), was mixed with the Au@AgNCs having an average edge length of 70.5 ± 3.2 nm (∼4 × 10^−11^ M). The mixed solution, including the dye and Au@AgNCs, was placed on a glass coverslip on the microscope. The Au@AgNCs were sedimented by spontaneous aggregation, and fell to the bottom; various assembly structures such as close-packed and ring-like structures were formed. Sedimentation of the Au@AgNCs was almost complete within an hour. The metal-induced fluorescence of the probe molecules near the Au@AgNCs was measured during sedimentation of the metal nanocubes (Scheme 1).

**Fig. 5:**
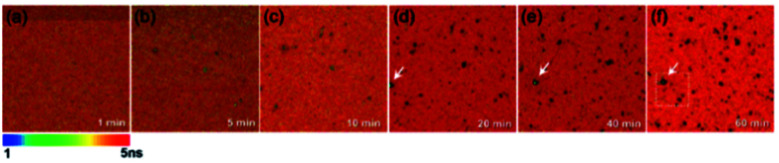
Fluorescence lifetime images of the mixture of dye molecules and Au@AgNCs during sedimentation for 60 min.

**Fig. 6:**
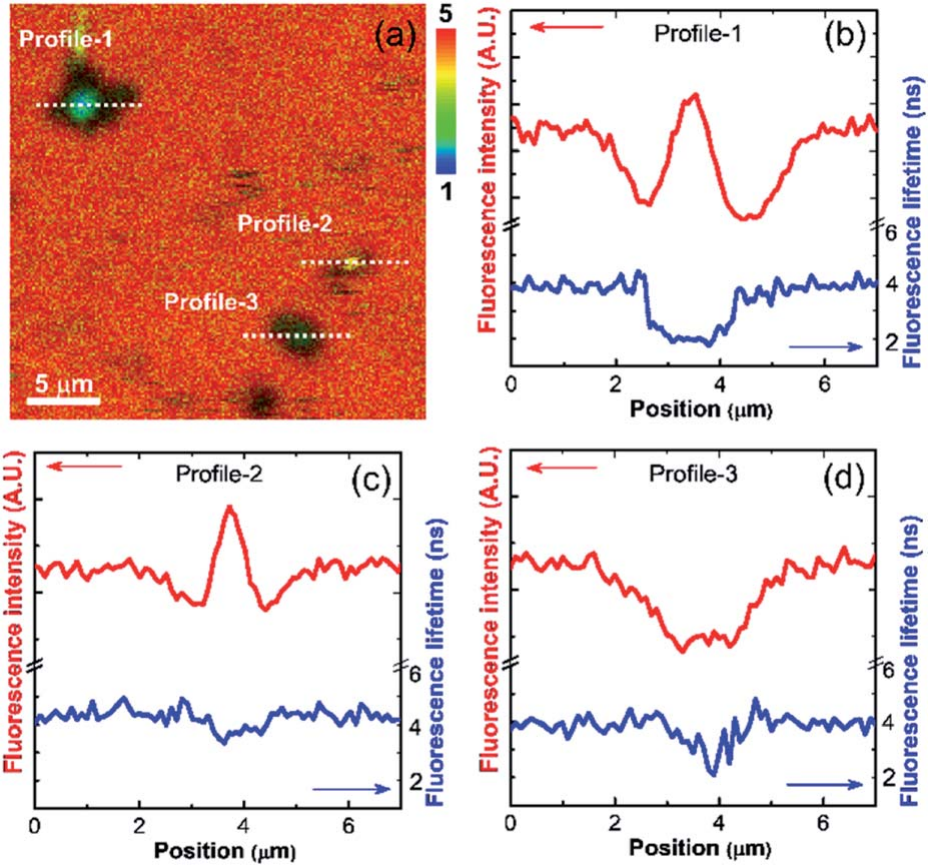
(a) Magnified fluorescence lifetime image of dye molecules near the Au@AgNCs aggregates in the dotted area of Fig. 5f. The rainbow scale bar is in nanosecond (ns) time units. (b–d) Line-profile of fluorescence intensity and lifetime for dotted cross-section of (a).

**Fig. 7:**
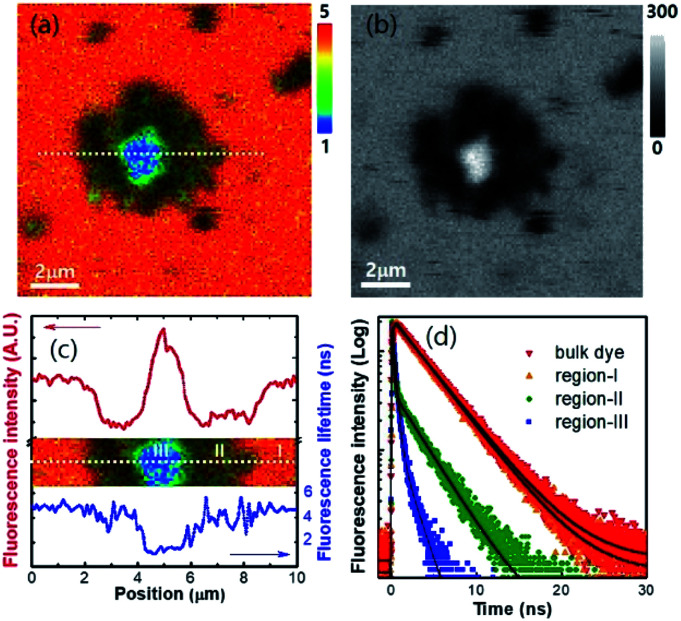
(a) Fluorescence lifetime images of dye molecules near the Au@AgNCs aggregates. The rainbow scale bar is in nanosecond (ns) time units. (b) Photon counting image of the corresponding FLIM image. (c) Line-profile of fluorescence intensity and lifetime in the dotted cross-section. (d) Time-resolved fluorescence decay curves observed at different local regions.

The sedimented aggregates quenched the dye emission, and were observed as dark spots compared to the surrounding dye emission (Fig. 5). On the other hand, interestingly, several assembly structures showed intensified emission at the center (indicated by an arrow). The magnified view shows a clear correlation between the fluorescence intensity and lifetime depending on the characteristics of the assemblies, as shown in Fig. 6. Profile-1 shows the interesting intensity and lifetime modulation profiles for an aggregate, whereas profile-3 shows simple quenching of both the intensity and lifetime. Profile-2, for a small aggregate, is similar to profile-1. For the fluorophores close to ring-like assembly systems, the site-dependent fluorescence properties could be classified into three distinct regions (Fig. 7). The intensity and lifetime of the probe molecules far from the Au@AgNC aggregates were unchanged (region-I, Fig. 7c). On the other hand, the metal nanocube aggregates reduced emissive radiation due to fluorescence quenching of the surrounding molecules through nonradiative charge trapping by the metal and radiation screening by metal nanocube itself,^11^ where the aggregates appeared as dark spots (region-II) compared to the surroundings (region-I).

The mean fluorescence lifetime was reduced to 2.61 ns compared to the lifetime of the bulk (*τ*_bulk_ = 4.00 ns). Notably, the internal void showed interesting fluorescence characteristics (region-III). The photon counting image clearly showed brighter emissions at the center of the assembly structures (Fig. 7b). The emission intensity was enhanced by 79% (Fig. 7c), while the fluorescence lifetime was reduced at the center (Fig. 7d and Table 1). According to previous reports, electromagnetic optical fields can accumulate in the void cavity of plasmonic metals.^24,25^ Therefore, the internal void may provide a specific circumstance enhancing the optical absorption of confined molecules. Meanwhile, the drastically reduced fluorescence lifetime indicates that the void cavity intensifies the radiative decay of the molecules (more intense emission), similar to the radiating plasmon model proposed in the literature.^26,27^

**Table 1:** Fluorescence lifetime of rhodamine 123 in proximity with Au@AgNCs aggregates^a^

Sample	*A*_1_ (%)	*τ*_1_ (ns)	*A*_2_ (%)	*τ*_2_ (ns)	〈*τ*〉 (ns)
Bulk dye	—	—	100	4.00 ± 0.03	4.00 ± 0.03
Region-I	—	—	100	3.86 ± 0.05	3.86 ± 0.05
Region-II	80	0.17 ± 0.03	20	3.15 ± 0.07	2.61 ± 0.07
Region-III	93	0.35 ± 0.05	7	1.60 ± 0.10	0.68 ± 0.10

a The time-resolved fluorescence intensity is described by: 
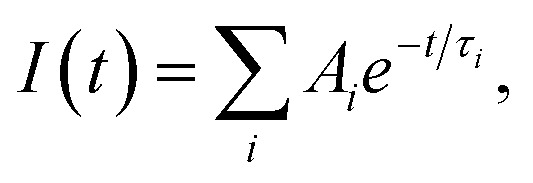
 where *I*(*t*) is the fluorescence intensity as a function of time, *A* is the amplitude as a normalized percentage, and *τ* is the fluorescence lifetime. The mean lifetime, 〈*τ*〉, is given by 
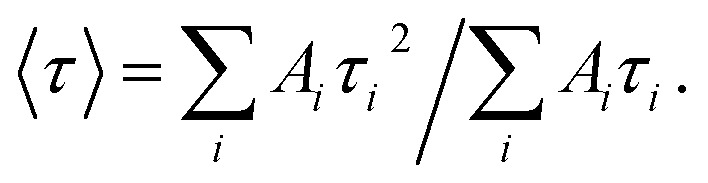

According to the theory, the intrinsic fluorescence lifetime (*τ*_0_) and quantum yield (*Φ*_0_) of a fluorophore can be described by, *τ*_0_ = 1/(*Γ* + *k*_nr_) and *Φ*_0_ = *Γ*/(*Γ* + *k*_nr_), respectively, where *Γ* and *k*_nr_ are the radiative and nonradiative decay rates.^11,28,29^ However, fluorophores near the metal surface have modified fluorescence lifetimes and quantum yields; *τ*_m_ = 1/(*Γ* + *Γ*_m_ + *k*_nr_) and *Φ*_m_ = (*Γ* + *Γ*_m_)/(*Γ* + *Γ*_m_ + *k*_nr_), respectively, where *Γ*_m_ is the additional term for the metal-coupled radiative decay rate.^11,28,29^ In this metal-fluorophore coupled system, the additional radiative *Γ*_m_ becomes a crucial term, which reduces the fluorescence lifetime (*τ*_m_) and increases the quantum yield (*Φ*_m_). Therefore, in the spontaneous assembly system of metal core–shell nanocubes, both accumulation of the optical field and the radiating plasmon model could act as driving forces leading to enhanced fluorescence in the internal void. Thus, it is revealed that the unique building block of Au@AgNCs has dual optical functions as a fluorescence quencher and fluorescence enhancement medium depending on the morphology of the aggregates.

Note that, AgNP aggregates always intensified emission on the metal surface without quenching process using identical imaging procedure (Fig. S2†). On the other hand, AuNPs always quench the fluorescence of surrounding molecules due to dominant nonradiative charge trapping by the Au metal (Fig. S2†). The Au@AgNCs only showed typical fluorescence depending on the shapes of aggregates. Although the voids were exhibited distinct fluorescence, further study needs to be conducted to elucidate the detailed mechanisms and effect of void radius.

## Conclusions

A simple and efficient methodology for the aqueous synthesis of uniform Au@AgNCs was developed, involving two sequential reactions, namely the preparation of Au NPs and stepwise Ag reduction on Au NPs to afford monodisperse Au@AgNCs. This core–shell structure was characterized by TEM and XRD. The size of the Au@AgNCs was modulated by changing the Ag shell thickness. The metal-induced fluorescence properties of probe molecules near the Au@AgNCs were measured during sedimentation of the Au@AgNCs. The unique ring-like building block of Au@AgNCs has dual optical functions as fluorescence quencher and fluorescence enhancement medium depending on the assembly regions. The Au@AgNCs aggregates quench the fluorescence of surrounding molecules due to nonradiative charge trapping by the metal, thereby appearing as dark regions compared to the surroundings, under microscopic analysis. On the other hand, very bright emission from the central internal void of the Au@AgNCs aggregates was observed, with a shortened fluorescence lifetime of the probe. It is expected that the Au@AgNC-enhanced fluorescence can be exploited in a variety of applications such as in the sensitive detection of molecules and metal ions.

## Conflicts of interest

There are no conflicts to declare.

## Supplementary Material

RA-009-C9RA05103A-s001

## References

[cit1] Lu F., Tian Y., Liu M., Su D., Zhang H., Govorov A. O., Gang O. (2013). Nano Lett..

[cit2] Liu X. L., Liang S., Nan F., Pan Y. Y., Shi J. J., Zhou L., Jia S. F., Wang J. B., Yu X. F., Wang Q. Q. (2013). Opt. Express.

[cit3] Langille M. R., Zhang J., Personick M. L., Li S., Mirkin C. A. (2012). Science.

[cit4] Zhao Y., Yang Y., Cui L., Zheng F., Song Q. (2018). Biosens. Bioelectron..

[cit5] Byers C. P., Zhang H., Swearer D. F., Yorulmaz M., Hoener B. S., Huang D., Hoggard A., Chang W. S., Mulvaney P., Ringe E., Halas N. J., Nordlander P., Linkand S., Landes C. F. (2015). Sci. Adv..

[cit6] Jiang H. L., Akita T., Ishida T., Haruta M., Xu Q. (2011). J. Am. Chem. Soc..

[cit7] Okuno Y., Nishioka K., Kiya A., Nakashima N., Ishibashi A., Niidome Y. (2010). Nanoscale.

[cit8] Haldar K. K., Kundu S., Patra A. (2014). ACS Appl. Mater. Interfaces.

[cit9] Lin S., Lin X., Liu Y., Zhao H., Hasi W., Wang L. (2018). Anal. Methods.

[cit10] Gong J., Zhou F., Li Z., Tang Z. (2012). Langmuir.

[cit11] Lakowicz J. R. (2001). Anal. Biochem..

[cit12] Deng W., Xie F., Baltar H. T., Goldys E. M. (2013). Phys. Chem. Chem. Phys..

[cit13] Pustovit V. N., Shahbazyan T. V. (2012). J. Chem. Phys..

[cit14] Coronado E. A., Encina E. R., Stefani F. D. (2011). Nanoscale.

[cit15] Jeong Y., Kook Y. M., Lee K., Koh W. G. (2018). Biosens. Bioelectron..

[cit16] Yang Z. L., Li Q. H., Ren B., Tian Z. Q. (2011). Chem. Commun..

[cit17] Luk'yanchuk B., Zheludev N. I., Maier S. A., Halas N. J., Nordlander P., Giessen H., Chong C. T. (2010). Nat. Mater..

[cit18] Liu X., Atwater M., Wang J., Huo Q. (2007). Colloids Surf., B.

[cit19] Jain P. K., Lee K. S., El-Sayed I. H., El-Sayed M. A. (2006). J. Phys. Chem. B.

[cit20] Sun Y., Xia Y. (2002). Science.

[cit21] Liu Y., Zhou J., Wang B., Jiang T., Ho H. P., Pettic L., Mormilec P. (2015). Phys. Chem. Chem. Phys..

[cit22] Kim D. K., Hwang Y. J., Yoon C., Yoon H. O., Chang K. S., Lee G., Lee S., Yi G. R. (2015). Phys. Chem. Chem. Phys..

[cit23] Tsao Y. C., Rej S., Chiu C. Y., Huang M. (2014). J. Am. Chem. Soc..

[cit24] Lee M.-J., Yang W.-G., Kim J. H., Hwang K., Chae W.-S. (2017). Microporous Mesoporous Mater..

[cit25] Baumberg J. J., Kelf T. A., Sugawara Y., Cintra S., Abdelsalam M. E., Bartlett P. N., Russell A. E. (2005). Nano Lett..

[cit26] Chae W.-S., Lee M.-J., Kim K., Hyun J. K., Jeon S. (2016). Appl. Phys. Lett..

[cit27] Aslan K., Leonenko Z., Lakowicz J. R., Geddes C. D. J. (2005). J. Fluoresc..

[cit28] Zhang Y., Aslan K., Previte M. J. R., Geddes C. D. (2007). Appl. Phys. Lett..

[cit29] Lakowicz J. R. , Principles of fluorescence spectroscopy, Springer, 3rd edn, New York, 2006, 978-0-387-46312-4

