# Supplementary material for: Understanding metal-enhanced fluorescence and structural properties in Au@Ag core–shell nanocubes
Source: RSC Adv. 2019 Sep 17;9(50):29232–7. doi: 10.1039/c9ra05103a (PMC9071844; doi:10.1039/c9ra05103a)
Supplement: RA-009-C9RA05103A-s001 [file RA-009-C9RA05103A-s001.pdf]

Electronic Supplementary Material (ESI) for RSC Advances.
This journal is © The Royal Society of Chemistry 2019

Electronic Supplementary Information (ESI)

Understanding Metal-Enhanced Fluorescence and Structural Properties in Au@Ag Core-shell Nanocubes

Dae-Woong Jung,[a, b] Jun Min Kim,[a, b] Hyung Joong Yun,[a] Gi-Ra Yi,[b] Jung Young Cho,[c] Haeun Jung,[d] Gaehang Lee,[*, a] Weon-Sik Chae,[*, e] and Ki Min Nam[*,d]

[a] *Korea Basic Science Institute, Daejeon, 34133, Republic of Korea*

[b] *Department of Chemical Engineering, Sungkyunkwan University, Suwon, 16419, Republic of Korea*

[c] *Korea Institute of Ceramic Engineering and Technology, Jinju 52851, Republic of Korea*

[d] *Department of Chemistry and Chemistry Institute for Functional Materials, Pusan National University, Busan 46241, Korea*

[e] *Analysis Research Division, Daegu Center, Korea Basic Science Institute, Daegu 41566, Republic of Korea*

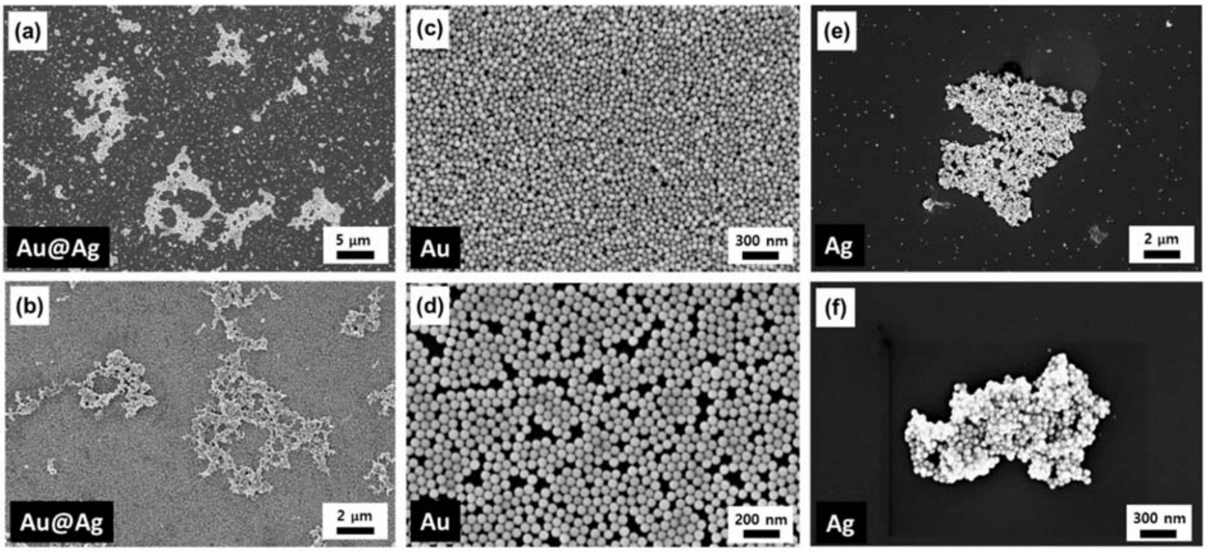

Fig. S1 SEM images of (a) and (b) Au@AgNCs, (c) and (d) AuNPs, (e) and (f) AgNPs on FTO after complete solvent evaporation.

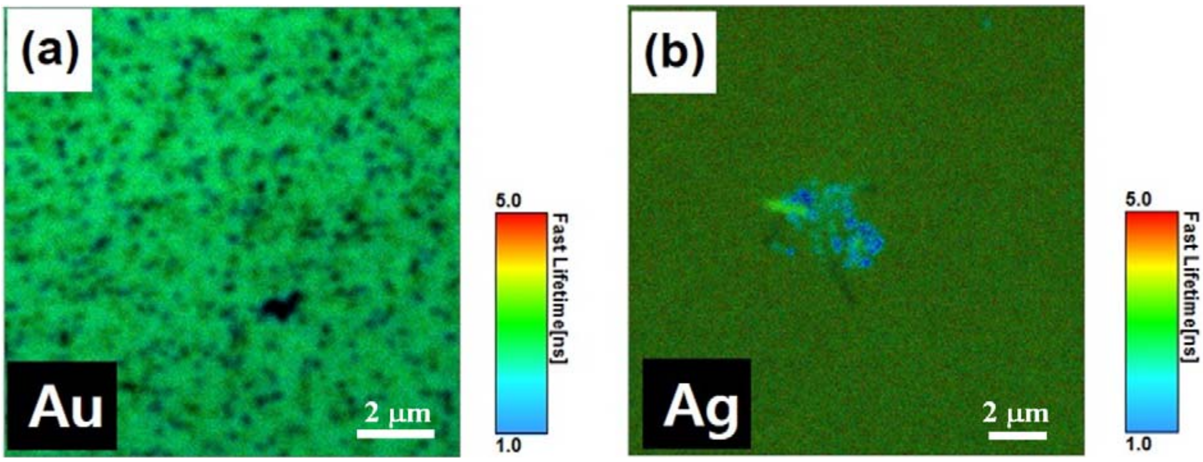

Fig. S2 Fluorescence lifetime images of (a) AuNPs and (b) AgNPs with dye molecules after sedimentation for 60 min using 470 nm excited laser.
